# The Necessity of Checking the Status and Performance of Methadone Maintenance Treatment Centers in Iran

**DOI:** 10.5812/ijhrba.10446

**Published:** 2013-03-12

**Authors:** Mohammad Davood Mohebi

**Affiliations:** 1 Department of Psychiatry, Zahedan University of Medical Sciences-Baharan Psychiatric Center, Zahedan, IR Iran

**Keywords:** Methadon, Substance-Related Disorders, Drug treatment Centers

Since 1941, when methadone was supplied as a synthetic drug to replace heroin consumption in Germany, nobody thought the drug will be used for treating and controlling opioid addiction, and as a way to prevent the outbreak of infections such as AIDS and hepatitis. Since 1980’s, with outbreak of HIV infection in European countries, especially through contaminated injections in heroin addicts, most attentions were attracted to oral methadone substitution. Although it caused numerous political and scientific challenges initially, using methadone as an alternative treatment for people addicted to opioid combinations has become quite common in Iran and most countries around the world, so that government backed general and specialized centers and clinics working in this field. Although there was some resistance in the UK at the beginning of widespread methadone treatment programs and some reasons were proposed by Russell Newcombe (1) about higher mortality rates of methadone compared to heroin and morphine, subsequent studies showed that risk of mortality in people being treated with methadone is 0.5% lower compared to pretreatment (2). In addition, suicide rates and risk of death due to car accidents are reported to be lower in these people compared to community mean (3).

Nowadays, it is quite obvious that methadone maintenance treatment (MMT) program reduces the abuse of opioid combinations mortality rate, committing crime, and risky behaviors resulting from their abuse as well. Moreover, this program enables patients to overcome social, family, and psychological harms caused by opioid dependency, and to improve general health of theirs and their families (2). In Iran, methadone maintenance treatment program began from 2010 as a legal program aimed at treating people addicted opioid combinations. The Program was in accordance with the protocol set by the Ministry of Health and Medical Sciences. Unlike most countries in which methadone is distributed through pharmacies with the supervision of responsible organizations, in Iran, only the centers that have license for opioid agonist treatment are allowed to prescribe methadone. Research has shown that in addition to benefits for the health of individuals and society, methadone maintenance treatment program, is economically useful and highly effective so that benefit-to-cost ratio is 4.4 to 1 and patient and society will enjoy these benefits (4). In the study of Keshtkaran et al. the incremental cost-effectiveness ratio is reported to be equal to $ 106382.39 in each case of AIDS prevention through methadone maintenance treatment (5).

Two of the most important problems that methadone treatment centers are dealing with include: risk of mortality caused by using methadone at the beginning of treatment due to the proximity of therapeutic dose and toxicity dose, and illegal distribution of methadone by clinics and turning it into black market trading (6). The main reason for the occurrence of this mortality is incongruence with treatment guidelines for increasing the dose of methadone during the induction and incidence of toxicity caused by it. Social stigma of addiction and patients’ concern will cause them not to refer to MMT centers on one hand, and tending towards obtaining methadone through illegal ways on the other hand, are the main factors in black market trading for methadone, which needs further studies. One of the most important challenges that MMT clinics and organizations associated with treating and controlling opioid dependency are facing is patients’ lack of resistance in methadone maintenance treatment program. Quitting MMT treatment program, along with recurrence increasing up to 50% during 1-3 months, leads to increasing mortality; that is, the mortality rate in patients quitting the treatment program is twice the rate of those staying in the program (4). Research has shown that properties of any curative instruction and features of each treatment unit play a significant role to achieve the main goals of MMT program. The most important factors in the success of the treatment and survival of patients in treatment program include: providing adequate dose of methadone, presence of a trained and experienced team, and trusting in the relationship between therapists and patients. In the study of Fakhraei et al, the average daily dose of methadone associated with the highest survival rate is reported to be 77.5 mg (7). In the study of Hosseini et al, doses higher than 125 mg have been associated with higher survival in treatment (8). In addition to the characteristics of treatment centers, personality characteristics of patients are also influential for achieving successful results. Concurrent presence of antisocial personality and concurrent use of cocaine have been reported to be related to quitting treatment program in a study (9). It seems that concurrent addiction to multiple drugs and high addiction rate need strategies beyond methadone maintenance treatment alone, because in such comorbidity, the rate of quitting MMT treatment program is high. Now that four years have passed since methadone maintenance treatment program began legally in Iran, it is necessary to evaluate the performance and characteristics of the country’s treatment centers with standard criteria, and to make decisions about their structure and how they should act. Obviously, independent and systematic evaluations by experienced research teams will be able to identify and analyze the deficiencies and limitations of MMT centers.
